# Novel Mechanisms of Sildenafil in Pulmonary Hypertension Involving Cytokines/Chemokines, MAP Kinases and Akt

**DOI:** 10.1371/journal.pone.0104890

**Published:** 2014-08-18

**Authors:** Tamas Kiss, Krisztina Kovacs, Andras Komocsi, Adrienn Tornyos, Petra Zalan, Balazs Sumegi, Ferenc Gallyas, Krisztina Kovacs

**Affiliations:** 1 Department of Anaesthesiology and Intensive Therapy, University of Pécs, Pécs, Hungary; 2 Department of Pathology, University of Pécs, Pécs, Hungary; 3 Heart Institute, University of Pécs, Pécs, Hungary; 4 Department of Biochemistry and Medical Chemistry, University of Pécs, Pécs, Hungary; 5 MTA-PTE Nuclear-Mitochondrial Research Group, Pécs, Hungary; 6 Szentágothai Research Center, University of Pécs, Pécs, Hungary; University of Giessen Lung Center, Germany

## Abstract

Pulmonary arterial hypertension (PH) is associated with high mortality due to right ventricular failure and hypoxia, therefore to understand the mechanism by which pulmonary vascular remodeling initiates these processes is very important. We used a well-characterized monocrotaline (MCT)-induced rat PH model, and analyzed lung morphology, expression of cytokines, mitogen-activated protein kinase (MAPK) phosphorylation, and phosphatidylinositol 3-kinase-Akt (PI-3k-Akt) pathway and nuclear factor (NF)-κB activation in order to elucidate the mechanisms by which sildenafil's protective effect in PH is exerted. Besides its protective effect on lung morphology, sildenafil suppressed multiple cytokines involved in neutrophil and mononuclear cells recruitment including cytokine-induced neutrophil chemoattractant (CINC)-1, CINC-2α/β, tissue inhibitor of metalloproteinase (TIMP)-1, interleukin (IL)-1α, lipopolysaccharide induced CXC chemokine (LIX), monokine induced by gamma interferon (MIG), macrophage inflammatory protein (MIP)-1α, and MIP-3α. NF-κB activation and phosphorylation were also attenuated by sildenafil. Furthermore, sildenafil reduced extracellular signal-regulated kinase (ERK)1/2 and p38 MAPK activation while enhanced activation of the cytoprotective Akt pathway in PH. These data suggest a beneficial effect of sildenafil on inflammatory and kinase signaling mechanisms that substantially contribute to its protective effects, and may have potential implications in designing future therapeutic strategies in the treatment of pulmonary hypertension.

## Introduction

Pulmonary arterial hypertension (PH) is a progressive and incurable disease that progresses to severe right heart failure. The disease is characterized by increase of the pulmonary vascular resistance due to obstructive proliferative changes in the lung microcirculation [1]. Our knowledge regarding the multiple pathological processes leading to the evolution of microvascular injury is still limited. Multiple lines of evidence support that vasoconstriction as well as inflammatory processes precede the remodeling of the pulmonary arterioles [2]. Endothelial dysfunction interferes at various levels with the microvascular injury characterized by medial hypertrophy, intimal proliferative changes, adventitial thickening with perivascular inflammatory infiltrates. These alterations as well as increased level of reactive oxygen species account for decreased apoptosis and increased proliferative vascular remodeling has significant role in the pathogenesis and progression of PH [3].

Our current therapeutic approach is based on application of vasodilator drugs despite that inflammation seems to be an important factor in the pathogenesis of PH [4].

Data from human studies as well as from monocrotaline (MCT) induced experimental rat PH model support that alveolar macrophages are the main source of cytokines and chemokines. The MCT treatment jeopardize pulmonary endothelial cells [5], [6] causing pulmonary artery smooth muscle (SM) hypertrophy with persistent pulmonary hypertension [7]. MCT not only injures the pulmonary arteries but also induces alveolar edema, alveolar septal cell hyperplasia, and occlusion of pulmonary veins [8], [9]. However, the pulmonary epithelium also produces these proinflammatory mediators, and lymphocytes and macrophages infiltrates as well as increased local expression of chemokines were found in the plexiform lesions of PH [10], [11].

Sildenafil, an inhibitor of phosphodiesterase type 5 (PDE-5) is widely used in the treatment of PH, and was demonstrated to improve exercise capacity, symptoms, and haemodynamics [12], [13]. However, the precise molecular mechanism of the protective effect of this drug is not completely understood. PDE-5 degrades the second messenger cGMP to GMP. Sildenafil by inhibiting PDE-5 increases intracellular cGMP level leading to smooth muscle cell (SMC) relaxation and produces beneficial effect on vascular remodeling and vasodilation through several pathways.

In a simple and widely accepted animal PH model, following two weeks of a single subcutaneous injection of MCT, rapid induction of severe pulmonary vascular disease consisting of pulmonary vascular remodeling and elevated pulmonary pressure can be observed with morphology similar to that observed in the human disease [14], [15].

In the present work using MCT induced rat PH, we investigated the protective role of sildenafil in the lung, and the mechanisms by which it contributed to attenuating the MCT induced inflammatory processes. We aimed to characterize changes in the cytokine network using a comprehensive array of 29 cytokines. Furthermore, we explored nuclear factor (NF)-κB activation, mitogen-activated protein kinases (MAPK) and the phosphatidylinositol 3-kinase-Akt (PI-3K)-Akt pathway.

## Materials and Methods

### Materials

Protease inhibitor cocktail, phosphatase inhibitor cocktail and MCT were purchased from Sigma Aldrich Co. (Budapest, Hungary). All reagents were of the highest purity commercially available.

### Solution protocols

MCT was dissolved in 0.5N HCl and the pH was adjusted to 7.4 using 0.5N NaOH.

The sildenafil solution was obtained by pulverizing 100 mg sildenafil tablets (Pfizer) dissolved to 0.2 mg/ml in drinking water. Animals were treated with freshly made solutions.

### Experimental protocol

The investigation conformed to the *Guide for the Care and Use of Laboratory Animals* published by the US National Institutes of Health, and was approved by the Animal Research Review Committee of the University of Pécs, Hungary. All animals were housed one or two per cage, under optimal laboratory conditions (controlled temperature, humidity and 12:12 h- light-dark cycles) with free access to water and standard rodent chow. The animals were randomly assigned into four groups (i) Six animals were allocated to Sham group (n = 6) receiving subcutaneous injection of isotonic saline (0.1 ml/kg) on day 0. (ii) Eight animals were allocated to Sham+SLD group (n = 8) receiving subcutaneous injection of isotonic saline (0.1 ml/kg) on day 0 and sildenafil (2 mg/kg per day, per os, in the drinking water) from day 0 to day 28. (iii) Eight animals were allocated to PH group (n = 8) receiving 60 mg/kg subcutaneous injection of MCT on day 0. (iv) Eight animals were allocated to PH+SLD group (n = 8) receiving 60 mg/kg subcutaneous injection of MCT on day 0 and sildenafil (2 mg/kg per day, per os, in the drinking water) from day 0 to day 28.

### Lung histology

Lungs were removed on day 28 under deep anesthesia, quickly blotted free of blood, weighed, and processed as required for histology and immunohistology. Lungs from 3 and 4 rats of control (Sham) and each of the other groups, respectively, were fixed in 6% formalin, embedded into paraffin and 5 µm thin sections were cut with a microtome. Sections were stained with haematoxylin–eosin and digital photos were taken. Average wall thickness of alveolar sac was determined at randomly chosen 25 different sites in each section by an expert who was blind to the experiment. Macrophages were counted in 5 non-overlapping high power fields (200×) in each sections by an expert who was blind to the experiment.

### Immunohistochemical staining

Slides were deparaffinized in xilene, rehydrated in graded ethanol series, and washed in distilled water. Heat induced epitope retrieval was performed by boiling the tissue sections in citrate buffer (HISTOLS Citrate Buffer, cat# 30010; Histopathology Ltd.) in a microwave oven at 750 W followed by cooling at room temperature for 20 minutes. Slides were washed in tris buffered saline (TBS) solution (pH = 7,6) followed by blocking of endogenous peroxidase (Peroxidase blocking, cat#30012, Histopathology Ltd.) for 10 minutes at room temperature. Slides were washed in TBS. Nonspecific sites were blocked (Background Blocking Protein Solution, cat#30013, Histopathology Ltd.) for 10 minutes at room temperature. Without washing, the following primary antibodies were applied: CD34: cat# 10097.10, clone: Q19-E, rabbit clonal antibody 1:200 dilution, Anti-NF-kB p65 (phospho S536) antibody (Abcam, cat#ab86299 in 1∶20 dilution). Incubation with the primary antibodies was performed for 1 hour at room temperature followed by washing in TBS. Secondary antibody (HISTOLS -R Detection System, anti-rabbit, Histopathology Ltd.) was applied for 30 minutes at room temperature followed by repeated washing in TBS. Sections were incubated with 3-amino-9-ethylcarbazol (HISTOLS -Resistant AEC Chromogen/Subsrtate System, cat# 30015.K, Histopathology Ltd.) or 3,3′-Diaminobenzidine (HISTOLS DAB Chromogen/Subsrtate System, cat#30014.K, Histopathology Ltd.), washed in distilled water, counterstained with haematoxylin followed by incubation in tap water. Negative control was incubated with antibody diluent instead of the primary antibody and applying anti-rabbit secondary antibody. Sections were then dehydrated, cleared in xilene and mounted with permanent mounting medium.

### Cytokine measurements

The cytokine array assay was performed on lung homogenates from 3 and 4 rats of control (Sham) and each of the other groups, respectively, using Rat Cytokine Array kit (R&D Systems; Biomedica Hungaria, Hungary). These arrays are based on binding between sample proteins and carefully selected capture antibodies spotted on nitrocellulose membranes. We examined tissue samples from all the 4 groups: Sham, Sham+ SLD, PH and PH+SLD groups. The array was performed as described by the manufacturer, similarly to our previous study [16]. Briefly, lung samples were homogenized in PBS with protease inhibitor. Triton X-100 was added to the final concentrations of 1%. After blocking the array membranes for 1 hour and adding the reconstituted detection antibody cocktail for another 1 hour at room temperature, the membranes were incubated with 1 ml of tissue homogenates containing 300 µg proteins at 2–8°C overnight on a rocking platform. After washing with buffer three times and addition of horseradish peroxidase-conjugated streptavidin to each membrane, the plates were exposed to a chemiluminescent detection reagent (Amersham Biosciences, Hungary), then were placed facing up to an X-ray film cassette. The array was run in duplicate sets. Developed films were scanned and analysed by densitometry. The pixel volumes of the bands were determined using the NIH ImageJ software, with the values in ratios of intensity.

### Immunoblotting

Lung tissue samples from 3 and 4 rats of control and each of the other groups, respectively, were homogenized in ice-cold isotonic Tris buffer (50 mM, pH 8.0) containing phosphatase and protease inhibitor cocktail (each 1∶1000; Sigma–Aldrich). After sonication, proteins were precipitated by trichloroacetate, washed three times with −20°C acetone, dissolved in Laemmli sample buffer, separated on 10% sodium-dodecil-sulfate (SDS)–polyacrylamide gels, and transferred to nitrocellulose membranes. After blocking for 2 hours with 3% nonfat milk in Tris-buffered saline, the membranes were probed overnight at 4°C with antibodies recognizing the following antigens: total-p38MAPK, phospho-p38 MAPK (Thr180/Tyr182), phospho-extracellular signal-regulated kinase (ERK)1/2 (Thr202/Tyr204), total ERK1/2, total GSK-3β, phospho-GSK-3β (Ser9), phospho-Akt (S473), total Akt, total-NF-κB, phospho-NF-κB (Ser536) (each 1∶500 dilution, Cell Signaling Technology). The membranes were washed six times for 5 minutes in Tris-buffered saline (pH 7.5) containing 0.2% Tween before addition of goat anti-rabbit horseradish peroxidase-conjugated secondary antibody (1∶3000 dilution; Bio-Rad). The protein bands were visualized with enhanced chemiluminescence (ECL) labeling using an ECL immunoblotting detection system (Amersham Biosciences). Developed films were scanned and the pixel volumes of the bands were determined using the NIH ImageJ software, with the values in ratios of intensity. Each experiment was repeated at least four times.

### Data analysis

The data are expressed as mean ± standard deviation. Data of alveolar sac thickness and densitometry were analyzed using the Kolmogorov-Smirnov normality test followed by the one-way Anova test and Bonferroni post hoc multiple comparison test. Differences were considered significant at p<0.05. Analyses were performed using IBM SPSS Statistics 20.

## Results

### Effects of sildenafil treatment on histological changes of the lung

**Figure 1:**
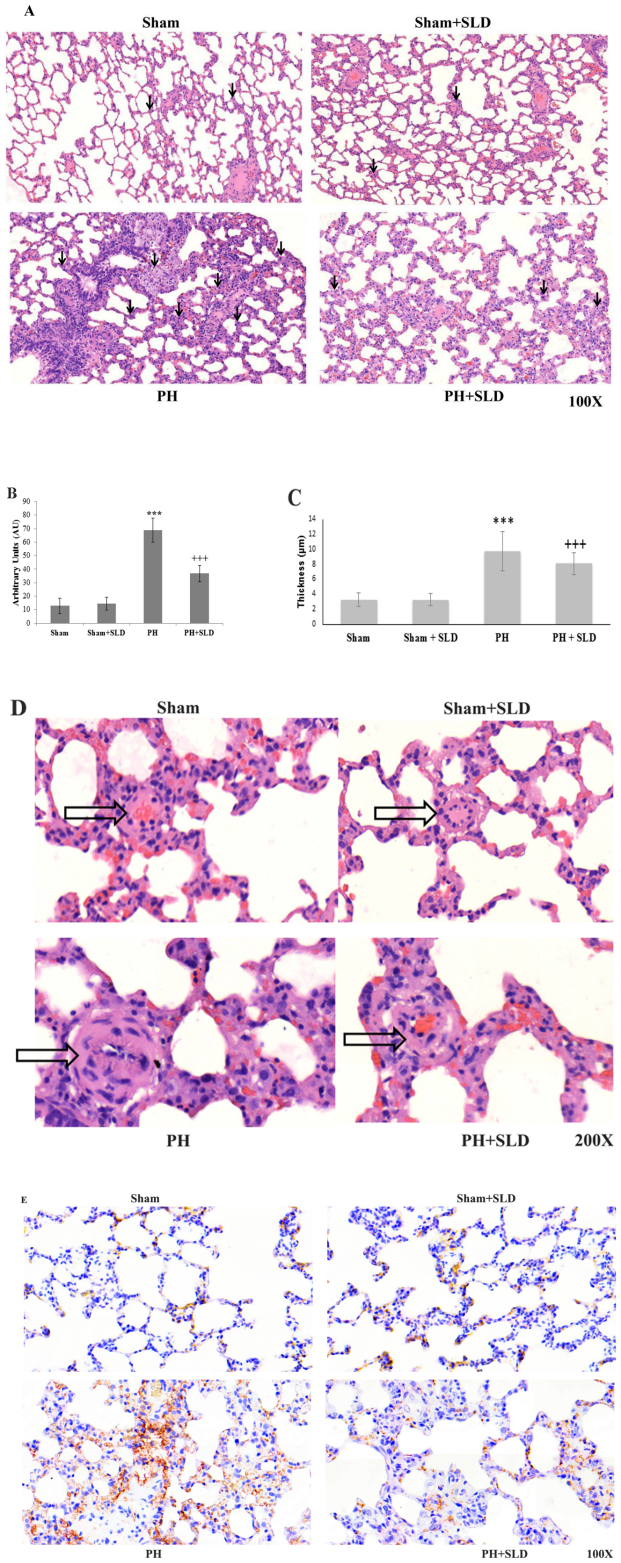
Effects of sildenafil treatment on histological changes of the lung. Formalin fixed lungs of untreated (Sham), sildenagfil (Sham+SLD), MCT (PH) and MCT plus sildenafil treated (PH+SLD) rats (n = 3–4) were subjected to histopathological analysis. Representative HE stained sections (A), macrophage infiltration (B), the mean wall thickness of alveolar sac (C), representative high power HE images of a blood vessel (D) and representative CD-34 immunohistochemistry images (E) indicating vascularization and vascular remodeling are presented. Macrophages and blood vessels are indicated by filled and empty arrows, respectively. Bar values are mean±S.E.M. *** significantly different from Sham group (p≤0.05), +++ significantly different from PH group (p≤0.05) by ANOVA, post hoc Bonferroni test.

Lung tissue of half of the animals in each group was subjected to histopathological study. Figure 1A demonstrates haematoxylin–eosin stained section of a Sham group rat; its alveolar sac and bronchioles with normal epithelium. There was a baseline level of macrophage infiltration (Figure 1B), the mean wall thickness of alveolar sac was 3.31±0.88 µm (Figure 1C), and vascular histology showed normal morphology (Figure 1D). CD-34 immunohistochemistry that indicates vascularization and vascular remodeling [17] showed sporadic weak positivity (Figure 1E). Sildenafil treatment alone did not cause any change in the aforementioned parameters (Figure 1). In MCT treated animals, the mean wall thickness of alveolar sac increased to 9.77±2.63 µm (p≤0.05) (Figure 1C) that was accompanied by a markedly increased macrophage infiltration (Figure 1A, B). We could also detect extensive vascular remodeling in the small pulmonary vessels- with intimal and medial hypertrophy of the muscular arteries and arterioles (Figure 1D,E). Sildenafil improved histological appearance of both the lung tissue (Figure 1A) and the blood vessels (Figure 1D,E), decreased macrophage infiltration (Figure 1B) as well as the alveolar sac wall thickness to 8.1±1.47 µm (p≤0.05) (Figure 1C).

### Effects of sildenafil treatment on cytokine expressions

**Figure 2:**
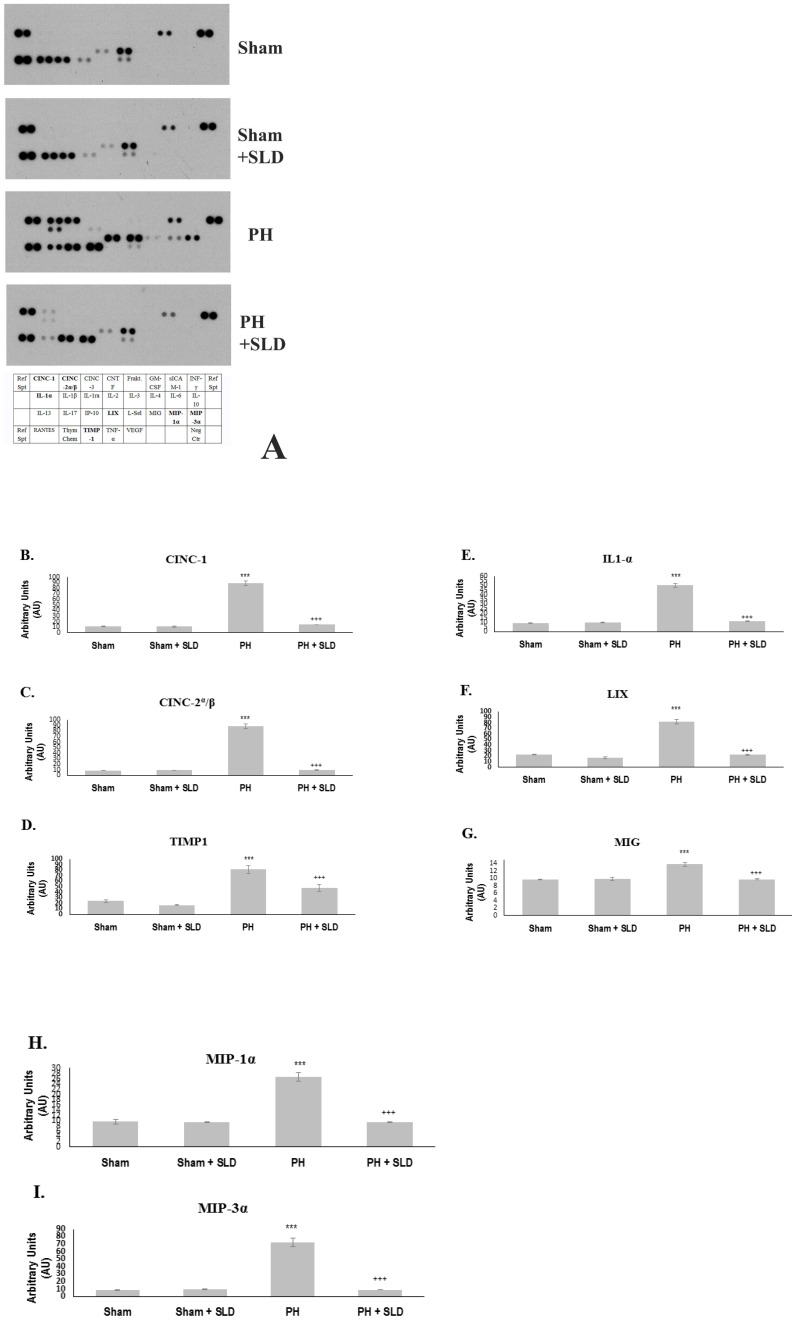
Effects of sildenafil treatment on cytokine expressions. Nitrocellulose based rat cytokine array assay was performed on lung homogenates of untreated (Sham), sildenagfil (Sham+SLD), MCT (PH) and MCT plus sildenafil treated (PH+SLD) rats (n = 3–4). Bar diagrams represent mean±S.E.M. pixel densities of the immunoblots. *** significantly different from Sham group (p≤0.05), +++ significantly different from PH group (p≤0.05) by ANOVA, post hoc Bonferroni test.

Since the histopathological study indicated inflammatory processes upon MCT treatment, we investigated the expression of 29 cytokines in our model. MCT significantly increased the expression of several cytokines (Figure 2) including the chemokines of the CXC group such as cytokine-induced neutrophil chemoattractant (CINC)-1, CINC-2α/β, lipopolysaccharide induced CXC chemokine (LIX) and monokine induced by gamma interferon (MIG). Expression of other chemoattractant proteins like macrophage inflammatory protein (MIP)-1α and MIP-3α was also increased. Levels of interleukin (IL)-1α, tissue inhibitor of metalloproteinase (TIMP)-1 were also elevated in MCT treated lungs. Sildenafil treatment attenuated the activation of these cytokines, as measured by a cytokine array system (Figure 2B–I). Sildenafil treatment alone did not affect cytokine expression significantly (Figure 2A).

### Effects of sildenafil treatment on NF-κB activation

**Figure 3:**
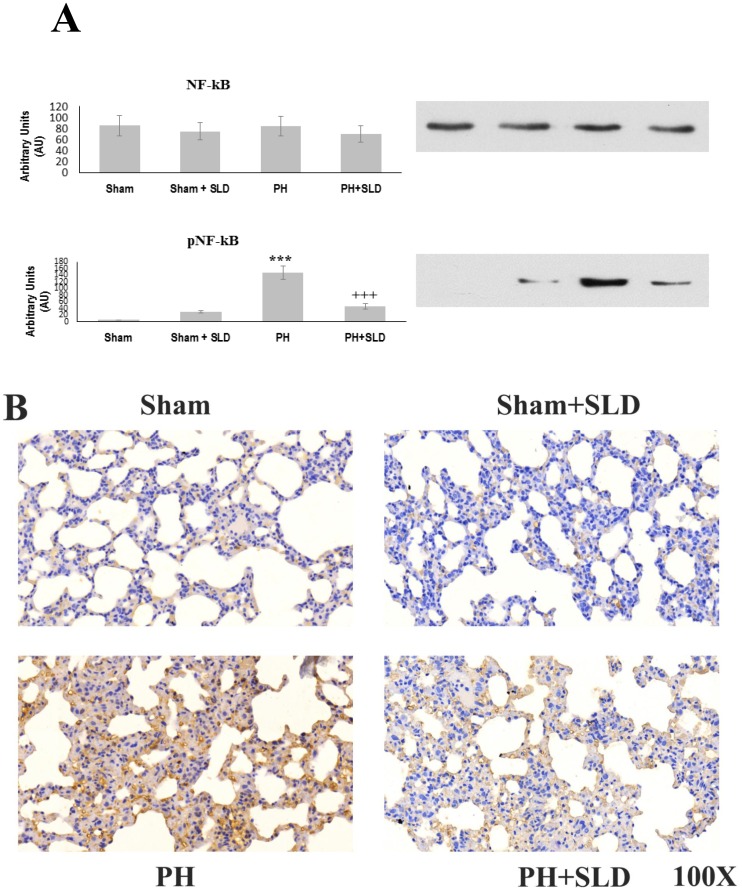
Effects of sildenafil treatment on NFκB activation and nuclear translocation. NFκB activation and nuclear translocation was assessed by immunoblotting (A) and immunohistochemistry (B) utilizing phospho-NF-κB p65 (A,B) and NF-κB (A) specific primary antibodies. Bar diagrams (A) of the mean pixel densities±S.E.M. and representative immunohistochemistry images (B) of 3 to 4 animals are presented. The phospho-NFκB p65 bands were normalized to the NFκB band. *** significantly different from Sham group (p≤0.05), +++ significantly different from PH group (p≤0.05) by ANOVA, post hoc Bonferroni test.

Expression of most the aforementioned cytokines that were significantly induced by MCT treatment are regulated by NF-κB transcription factor. Therefore, we determined NF-κB activation by immunoblotting and immunohistochemistry utilizing phospho-NF-κB p65 specific primary antibody among the various groups. Under our experimental conditions, none of the treatments affected expression level of NF-κB. On the other hand, we observed increased phosporylation thereby activation of NF-κB [18] in MCT treated lung tissues, which was diminished in sildenafil treated animal lungs (Figure 3A). Phospho-NF-κB p65 immunohistochemistry showed sporadic patchy weak background positivity in the Sham group. A very few of the nuclei demonstrated strong positive staining indicating nuclear translocation of NF-κB. NF-κB activation and nuclear translocation was basically identical to control among the sildenafil treated animals. MCT treatment caused a massive activation and nuclear translocation of NF-κB that was significantly diminished by sildenafil treatment (Figure 3B).

### Effect of sildenafil on PI-3K-Akt and MAPK signaling pathway

NF-κB activation is regulated by various kinase signaling pathways including PI-3K-Akt and MAPK pathways [19]. Therefore, we assessed the activation of these pathways by immunoblotting utilizing phosphorylation-specific primary antibodies.

**Figure 4:**
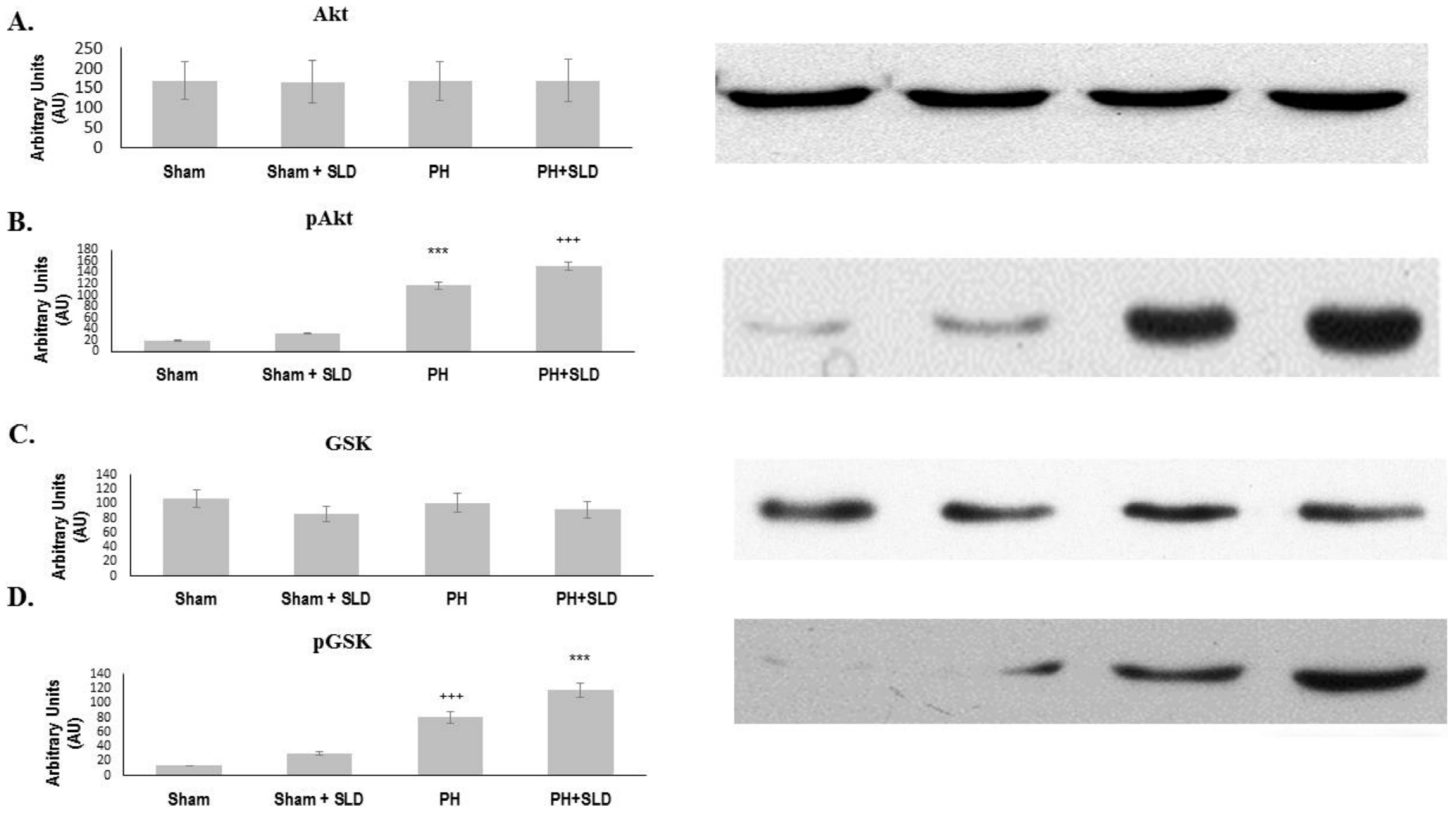
Effect of sildenafil on Akt activation. Effect of Sildenafil and MCT treatment on Akt expression (A) and activation (B) as well as on GSK-3β expression (C) and phosphorylation (D) was determined by immunoblotting utilizing protein- and phosphorylation specific primary antibodies. The bar diagrams represent pixel volumes±S.E.M. of pAkt and pGSK-3β bands. The bands were normalized to the appropriate Akt and GSK-3β bands. *** significantly different from Sham group (p≤0.05), +++ significantly different from PH group (p≤0.05) by ANOVA, post hoc Bonferroni test. Phospho-Akt and GSK-3β are denoted as pAkt and pGSK-3β.

Expression of Akt or its downstream target, glycogen synthase kinase (GSK)-3β was not affected by any of the treatment. The phosphorylation thereby activation of Akt was not detectable in control lung tissues (Figure 4). Sildenafil alone slightly increased Akt activation, although, it did not reach the level of statistical significance. MCT treatment significantly increased Akt phosphorylation that was further enhanced by sildenafil administration tissues (Figure 4). The phosphorylation of Akt's downstream target GSK-3β showed similar patter among the different groups as that of Akt verifying Akt activation (Figure 4).

**Figure 5:**
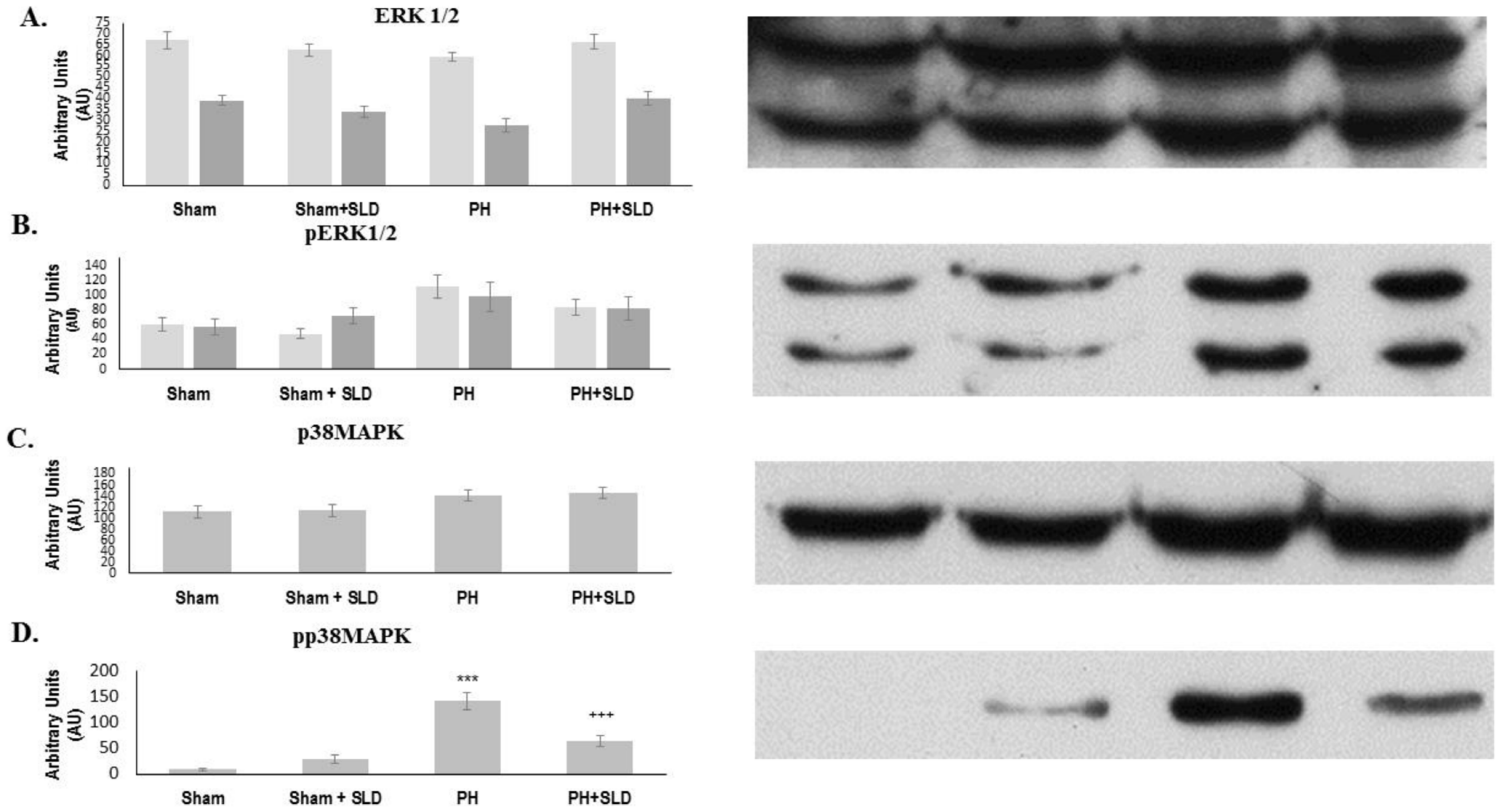
Effect of sildenafil on ERK1/2 and p38 MAPK activation. Effect of Sildenafil and MCT treatment on ERK 1 and 2 expression (A) and activation (B) as well as on p38 MAPK expression (C) and phosphorylation (D) was determined by immunoblotting utilizing protein- and phosphorylation specific primary antibodies. The bar diagrams represent pixel volumes±S.E.M. of pERK1/2 and pp38 MAPK bands. The bands were normalized to the appropriate ERK1/2 and p38 MAPK bands. *** significantly different from Sham group (p≤0.05), +++ significantly different from PH group (p≤0.05) by ANOVA, post hoc Bonferroni test. Phospho-ERK1/2 and p38 MAPK are denoted as pERK1/2 and pp38 MAPK.

Since cGMP was reported to regulate ERK1/2 and p38 among the MAPKs [20], we studied the effect of MCT and sildenafil on them. Expression of ERK1, 2 and p38 was not affected significantly by any of the treatment. In the control lungs with or without sildenafil treatment, a slight phosphorylation of ERK1/2 was observed (Figure 5). In MCT treated lungs a moderate activation of ERK1/2 occurred that was slightly decreased by sildenafil treatment, however, none of these changes reached the level of statistical significance (Figure 5). The p38 MAPK activation/phosphorylation in control lungs was undetectable that was slightly elevated by sildenafil treatment. A robust activation of p38 MAPK was observed when MCT induced PH occurred that was significantly diminished by sildenafil (Figure 5).

## Discussion

Vasodilators - including sildenafil, a PDE-5 inhibitor - are widely used to treat pulmonary arterial hypertension. Clinical data are supporting the benefits of this treatment in terms of improvement of symptoms, exercise capacity, haemodynamics, and possible outcome [21]. However, it is still unclear whether sildenafil affects microvascular injury via mechanisms that are independent from or additional to vasodilation. Increased pulmonary arterial pressure in PH results from a combination of pulmonary vasoconstriction, vascular remodelling, inflammation and, in some cases, in situ thrombosis. Elevated circulating levels of the proinflammatory cytokines IL-1β, IL-6, macrophage inflammatory protein-1α, and P-selectin have been observed in patients [22], [23]. A role for inflammation in the development of IPAH is supported by enhanced pulmonary expression of various cytokines and chemokines, such as fractalkine and their association with inflammatory cell infiltrates in severe PAH [24], [25]. In the MCT model, besides thickened, obstructed blood vessels and increased CD-34 immunoreactivity, we observed increased macrophage infiltration and increased wall thickness of aleolar sacs. Former findings reflect to vascular remodeling and vascularization, while the latter ones are due to inflammatory processes. These findings are in line with alterations described in earlier studies [26], [27]. Sildenafil's ability to ameliorate all the aforementioned pathological changes, suggested that it possesses anti-inflammatory characteristics. Anti-inflammatory effects of sildenafil were controversial in the earlier studies [28], [29] however recent studies in different models [30], [31] showed findings, which are in line with our results.

We found that sildenafil treatment significantly reduced the MCT induced expression of a number of cytokines and chemokines in lung homogenates by using a rat cytokine array. Among them, CINC-1 and CINC-2α/β have neutrophil chemotactic activity and promote neutrophil-facilitated cell damage in the lungs [32], [33]. In previous studies using an acute lung injury model, serum CINC-1 levels correlated with the elevated lung CINC-1 levels suggesting that circulating CINC-1 levels could be used as an early marker for the subsequent development of organ inflammation and injury [34]. LIX is a chemokine belonging to the CXC chemokine group, produced by type-II alveolar epithelial cells and NF-κB regulates its expression [35]. LIX facilitates neutrophil recruitment which can promote oxidative stress induced damages [36]. Similarly, MIG is another overexpressed, effective chemo-attractant for mononuclear cells from the CXC chemokine group which can also contribute to cell death [37]. MIP chemokines released from macrophages activate granulocytes and lymphocytes and enhance the synthesis of proinflammatory cytokines such as IL-1, IL-6 and tumor necrosis factor (TNF)-α, which play a crucial role in the pathomechanism of PH. Fartoukh et al. demonstrated increased MIP-1α mRNA expression in human lung biopsy specimens of PH patients [22]. Humbert et al showed slight increases in both IL-1 and IL-6 serum concentrations in severe PH patients [23]. These cytokines produced mainly by activated macrophages, neutrophils, epithelial and endothelial cells initiate inflammatory responses and oxidative stress [38], [39].

Most of the aforementioned cytokines' and chemokines' expression is regulated by NF-κB, which was reported to activate a number of cytokine and growth factor genes associated with PH [40], [41]. NF-κB is retained in the cytoplasm in its inactive form by its inhibitor, I-κB, which is subject to phosphorylation mediated degradation. A number of signaling kinases including MAPKs phosphorylate thereby activate I-κB kinase. After removal of I-κB, nuclear localization signal of NF-κB becomes unmasked, it translocates to the nucleus where it is activated by phosphorylation and acetylation to stimulate NF-κB dependent gene expression [42]. Accordingly, we confirmed our cytokine array findings by demonstrating that sildenafil attenuated MCT induced NF-κB activation. We performed NF-κB immunohistochemistry to identify the cells responsible for the cytokine and chemokine production, and in accord with previous reports by others [38], [39] we found that endothelial and epithelial cells as well as the infiltrating inflammatory cells all contributed to this process. A previous report [43] highlighted the relevance NF-κB activation in PH patients, while NF-κB's direct inhibition protected against the development of MCT induced PH [44]. Besides the aforementioned cytokines and chemokines, NF-κB regulates matrix metalloproteinase (MMP) expression too. Most probably, the increased TIMP-1 expression we observed developed as a compensatory mechanism [45] for the MCT induced MMP expression.

PI-3K–Akt signaling plays an essential role in regulating a number of cellular processes, including cellular growth and proliferation, migration and cell survival. PI-3K and Akt proteins are expressed constitutively in cultured vascular smooth muscle cells (VSMCs). Activated Akt has been found in vascular remodeling after vascular injury accompanied by VSMC proliferation [46]. Activation of Akt is tightly coupled to proliferation of VSMCs involved in vascular remodeling [47]. Pulmonary arterial smooth muscle cell (PASMC) phenotype switching, which is characterized by changes in smooth muscle (SM)-specific gene expression, contributes to vascular remodeling in PH. In addition, it has been shown that the transcription of SM-specific genes is modulated by cytoskeleton rearrangement. PI-3K-Akt pathway plays an important role in the modulation of PASMCs cytoskeleton rearrangement and phenotype switching [48]. The PI-3k-Akt pathway activates endothelial nitric oxide synthase (eNOS), and inactivates GSK-3β. eNOS activation can promote the vascular smooth muscle cell relaxation, and the inhibition of GSK-3β can prevent mitochondrial permeability transition which would lead to necrotic cell death [49]. In our PH model, Akt activation and GSK-3β inactivation may be interpreted as an unsuccessful compensatory mechanism against the oxidative and inflammatory damage induced by the MCT treatment. When augmented by sildenafil it could contribute to overcome the harmful consequences of the MCT treatment.

The MAPKs are a family of central signaling molecules that respond to numerous stimuli by phosphorylating a variety of substrates including transcription factors, enzymes, and other kinases, thereby orchestrating cellular proliferation, differentiation, survival, apoptosis and inlammatory processes. Also, the remodeling of vasculature had been related to MAPKs [50]–[52]. Inhibition of the downstream ERK1/2 signaling pathway activation prevents pulmonary vascular remodeling, elevated right ventricular pressure and improves right ventricular hypertrophy in MCT- and chronic hypoxia-induced PH model [53], [54]. Zeng et al showed that the phosphorylation levels of ERK1/2 are significantly increased in MCT induced PH rat model and sildenafil treatment can inhibit the increased expression of ERK phosphorylation in this model [50]. Fibroblasts in pulmonary arteries express all four isoforms of p38 MAPK [55]. Using the p38 MAPK inhibitor in the chronic hypoxic and MCT model resulted in lower right ventricular systolic pressure and right ventricular hypertrophy in the drug treated animals. Lu et al found an increase in p38 MAPK activity in MCT-treated rat lungs, and demonstrated that the production and effect of many of the potential inflammatory mediators in PH were associated with activation of the p38 MAPK signaling pathway. A selective p38 MAPK inhibitor significantly attenuated the expression of inflammatory cytokines in this study [49]. In our experimental model, pulmonary hypertension activates ERK1/2 and p38 MAPK, but sildenafil partially suppresses their activation ultimately preventing the progression of vascular remodeling and inflammation in PH that is completely in line with the aforementioned findings.

In conclusion, in our experimental model, elevated cGMP level by sildenafil most probably via activating a protein kinase G induced a crosstalk between kinase signaling systems [20]. It results in sufficient enhancement of Akt activation to overcome the pathologic remodeling processes induced by the MCT treatment, decrease of MAPK activation and NF-κB nuclear translocation. Latter, in turn, causes decreased cytokine and chemokine production reducing inflammatory cell infiltration and edema formation. All these data suggest a novel mechanism for the beneficial effects of sildenafil in PH that is independent and additional to its main vasodilatory effect.
